# Integrating multiple molecular sources into a clinical risk prediction signature by extracting complementary information

**DOI:** 10.1186/s12859-016-1183-6

**Published:** 2016-08-30

**Authors:** Stefanie Hieke, Axel Benner, Richard F. Schlenl, Martin Schumacher, Lars Bullinger, Harald Binder

**Affiliations:** 1 Institute for Medical Biometry and Statistics, Faculty of Medicine and Medical Center - University of Freiburg, Stefan-Meier-Str. 26, Freiburg, 79104 Germany; 2 Freiburg Center for Data Analysis and Modeling, University Freiburg, Eckerstr. 1, Freiburg, 79104 Germany; 3 Division of Biostatistics, German Cancer Research Center, Im Neuenheimer Feld 280, Heidelberg, 69120 Germany; 4 Department of Internal Medicine III, University Hospital of Ulm, Albert-Einstein-Allee 23, Ulm, 89081 Germany; 5 Institute of Medical Biostatistics, Epidemiology and Informatics, University Medical Center Johannes Gutenberg University Mainz, Obere Zahlbacher Str. 69, Mainz, 55131 Germany

**Keywords:** Acute myeloid leukemia, Multiple genome-wide data sets, Risk prediction, Multivariable model, Boosting, Time-to-event endpoint

## Abstract

**Background:**

High-throughput technology allows for genome-wide measurements at different molecular levels for the same patient, e.g. single nucleotide polymorphisms (SNPs) and gene expression. Correspondingly, it might be beneficial to also integrate complementary information from different molecular levels when building multivariable risk prediction models for a clinical endpoint, such as treatment response or survival. Unfortunately, such a high-dimensional modeling task will often be complicated by a limited overlap of molecular measurements at different levels between patients, i.e. measurements from all molecular levels are available only for a smaller proportion of patients.

**Results:**

We propose a sequential strategy for building clinical risk prediction models that integrate genome-wide measurements from two molecular levels in a complementary way. To deal with partial overlap, we develop an imputation approach that allows us to use all available data. This approach is investigated in two acute myeloid leukemia applications combining gene expression with either SNP or DNA methylation data. After obtaining a sparse risk prediction signature e.g. from SNP data, an automatically selected set of prognostic SNPs, by componentwise likelihood-based boosting, imputation is performed for the corresponding linear predictor by a linking model that incorporates e.g. gene expression measurements. The imputed linear predictor is then used for adjustment when building a prognostic signature from the gene expression data. For evaluation, we consider stability, as quantified by inclusion frequencies across resampling data sets. Despite an extremely small overlap in the application example with gene expression and SNPs, several genes are seen to be more stably identified when taking the (imputed) linear predictor from the SNP data into account. In the application with gene expression and DNA methylation, prediction performance with respect to survival also indicates that the proposed approach might work well.

**Conclusions:**

We consider imputation of linear predictor values to be a feasible and sensible approach for dealing with partial overlap in complementary integrative analysis of molecular measurements at different levels. More generally, these results indicate that a complementary strategy for integrating different molecular levels can result in more stable risk prediction signatures, potentially providing a more reliable insight into the underlying biology.

**Electronic supplementary material:**

The online version of this article (doi:10.1186/s12859-016-1183-6) contains supplementary material, which is available to authorized users.

## Background

When molecular measurements are obtained from patients in a clinical cohort by high-throughput techniques, the number of covariates is typically much larger than the number of observations. There are established statistical techniques for linking measurements from a single molecular level to clinical endpoints such as survival. In particular, techniques for regularized estimation of regression models, such as the lasso [1] or componentwise likelihood-based boosting [2] can automatically select potentially important molecular entities.

In more recent studies, microarray and sequencing technologies are used to measure a large number of patients’ characteristics at different molecular levels simultaneously. For example, the data that motivated us to develop an integrative analysis strategy comes from a study on acute myeloid leukemia (AML) patients with single nucleotide polymorphism (SNP) microarray measurements [3] and microarray-based gene expression profiling (GEP) data [4, 5]. Corresponding to what might be considered the natural biological order, we propose a sequential complementary strategy in the following as a tool for integrative data analysis, which first extracts information from the SNP data, and then looks for additional prognostic information in the gene expression measurements. Ideally, the knowledge obtained from the SNP data should help to stabilize selection of genes for a prognostic signature based on the microarray-based GEP data.

Such an integrative analysis is complicated by having a partial overlap, i.e. not all molecular levels are measured for all patients. For example in our AML application, there are some patients with measurements available from both genome-wide measurement platforms, but some patients have measurements from only one single molecular level, either from the microarray-based GEP or from the SNP microarray. An indication that this is a more widespread problem is provided, e.g. by the data available in the Cancer Genome Atlas (TCGA) (https://tcga-data.nci.nih.gov/tcga/). Due to a multitude of reasons (assay failure, sample quality issues, changes in measurement platforms, etc.), not every type of measurement is available for each individual. As a straightforward complete case analysis would discard much of the available information, partial overlap has to be addressed before an integrative analysis can be performed.

We propose a corresponding imputation approach that fits with our sequential integrative analysis strategy. In particular, we will show that our sequential strategy does not require imputation at the SNP level, but only imputation of a linear predictor that based on the SNPs. Estimation for the prognostic models, i.e. the linear predictors, as well as for an imputation regression model will be performed by componentwise likelihood-based boosting [2, 6].

While a multitude of statistical techniques have been developed for building models that predict a clinical endpoint based on high-dimensional data from a single molecular level, combined analysis of multiple genome-wide data sets relating to clinical endpoints has received less attention. Integrative analysis of several platforms using multivariable techniques such as clustering [7] or principle components [8, 9] is often performed for assessing the relations between various levels but without taking a clinical endpoint into account. In contrast, the proposed sequential complementary strategy directly links the different molecular levels of information to a clinical endpoint, such as time to death, while taking established clinical predictors into account.

The paper is organized as follows: The following section introduces two applications, where a prognostic model is wanted for patients with acute myeloid leukemia (AML), and then details the proposed sequential complementary strategy, including a stepwise estimation procedure and an imputation approach. In the results section, the sequential complementary strategy is illustrated for SNP and microarray-based gene expression data in the first AML application and for methylation and gene expression sequencing data in the second AML application. In the subsequent section, the results are discussed and more general concluding remarks are provided.

## Methods

### Acute myeloid leukemia data

Acute myeloid leukemia (AML), the most common acute leukemia in adults, represents a genetically heterogeneous disease with respect to clinical outcome, such as time to relapse or death. Currently, several clinically relevant molecular markers are known that characterize AML at the molecular level [3]. An integrative analysis of molecular data from different molecular platforms might help to identify additional molecular subgroups relevant for the prognosis of AML patients. This is of particular importance as current AML molecular-based classification based on single molecular levels are not robust enough to predict the prognosis for AML patients, due to the heterogeneity within AML [10].

In the two motivating data examples considered in the following, the time-to-event clinical endpoint of interest is survival (relapse free survival (RFS), i.e. time to relapse or death from any cause, whatever happens first, in a first application, and overall survival in a second application). In the first application, measurements from two different molecular levels, single nucleotide polymorphism (SNP) information from microarrays [3] and microarray-based gene expression (GEP) data [4, 5] are available for developing a prognostic signature. The data are included in Additional files 1, 2 and 3. Specifically, there are 390443 SNP features for 308 AML patients and 19088 gene expression features for 319 AML patients, with an overlap of 26 patients where both types of measurements are available. During follow-up time, 154 AML patients with SNP measurements and 196 AML patients with GEP measurements had a relapse or died. As a benchmark for prognostic models, the established predictors age, white blood cell count (WBC), somatically acquired mutations in nucleophosmin 1 (*NPM*1) and fms-related tyrosine kinase 3 internal tandem-duplication (*FLT*3−*ITD*) genes as well as a cytogenetic risk group factor are available for all AML patients.

The second application, considers methylation and gene expression sequencing data as well as clinical information from the Cancer Genome Atlas (TCGA). Data sets were not available for all individuals on all platforms because of same availability, assay failure and quality issues. DNA methylation information for 194 patients were generated from the Illumina Infinium HumanMethylation450 BeadChip for bisulfite-treated DNA. There are measurements of methylation status for 485577 CpG sites for each patient. After pre-processing steps, 396065 methylation features remain. Twelve samples were excluded due to missing outcome data. Of these 182 AML patients, 118 AML cases died during their follow-up. In addition to methylation measurements, there are gene expression sequencing data (RNA-seq) for 179 AML cases, comprising 20442 RNA-seq measurements per patient. Ten of 179 AML cases were excluded due to missing outcome information. As mentioned above, death is the event of interest. This event was observed in 108 out of 169 AML cases. Since our focus lies on the integrative analysis of molecular measurements at different levels while taking clinical predictors into account, two clinical covariates (sex and age at initial pathological diagnosis) available for all AML cases are investigated. In total, there is an overlap of 166 AML cases with information from both types of molecular levels. The data can be downloaded directly from the TCGA website (https://tcga-data.nci.nih.gov/tcga/).

### Sequential complementary strategy

In the following, we will propose an approach for integrative analysis in both applications above, the first considering microarray-based gene expression profiling (GEP) and SNP data, and the second considering gene expression sequencing (RNA-seq) and methylation data, to illustrate the general applicability of our proposal. Yet, notation and explanation will focus on the combination of GEP and SNP data (first AML application) in the following, for simplicity.

**Fig. 1:**
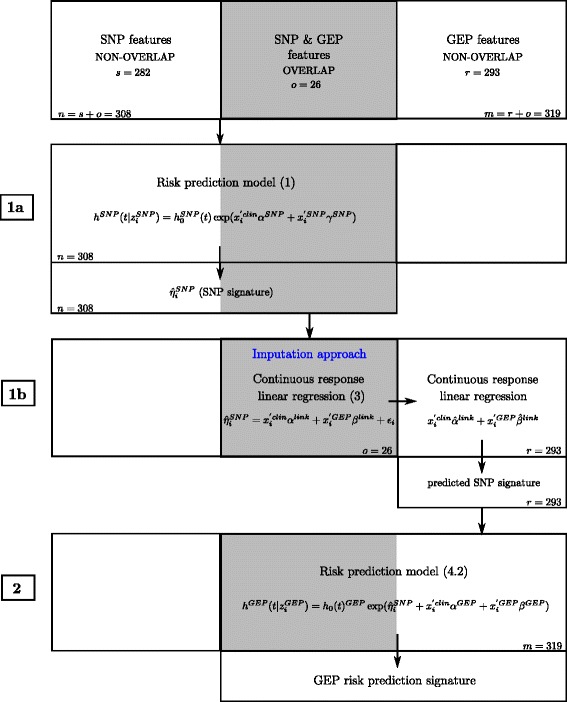
Flowchart describing the sequential complementary strategy based on a stepwise procedure (notation focuses on the first AML application combining GEP and SNP data). Established clinical predictors (*clin*) that need to be adjusted for are considered as mandatory in the stepwise procedure based on componentwise likelihood-based boosting. Important clinical predictors are available for all AML cases. The SNP signature ($\hat {\eta }_{i}^{SNP}$) including the known SNP signature (overlap samples) from model (1) and the predicted SNP signature (non-overlap samples) considering continuous response linear regression (3) as prediction technique is incorporated as fixed offset in model (2) for the microarray-based GEP data

Specifically, we propose a sequential complementary strategy for an integrative analysis of different molecular levels with potentially partial overlap in the biological samples. We first provide two risk prediction models, for the SNP data and the GEP data respectively, followed by an imputation approach for dealing with partial overlap, and finally the technical details of model fitting and a brief discussion of the order in which SNP and GEP data are considered for modeling. A flowchart describing the sequential complementary strategy based on the stepwise procedure and the imputation approach is given in Fig. 1.

Censored time to event data, e.g. as in the time to relapse AML application above, are typically given as triples (*t*_*i*_,*δ*_*i*_,*z*_*i*_), *i*=1,…*n*, where *t*_*i*_ is the observed time for an individual, which is given by *t*_*i*_= min(*T*_*i*_,*C*_*i*_), with event time *T*_*i*_ and censoring time *C*_*i*_. The event indicator *δ*_*i*_=*I*(*T*_*i*_≤*C*_*i*_), with indicator function *I*(.), takes value 1 if its argument is true, and 0 otherwise. The covariate vector *z*_*i*_=(*z*_*i*1_,…,*z*_*ip*_)^′^ comprises covariates known at baseline. We assume that it can be partitioned into a vector $x_{i}^{clin}$ of *d* clinical covariates, a vector $x_{i}^{SNP}$ of *k* SNP covariates, and a vector $x_{i}^{GEP}$ of *q* gene expression covariates. In the following, the vectors $z_{i}^{SNP}$ and $z_{i}^{GEP}$ are assumed to comprise the SNP and microarray-based GEP covariates respectively, in addition to the clinical covariates.

A common regression model for time to event data is the Cox proportional hazard model [11]. For the SNP data, the proposed model is given by

1 $$  \begin{aligned} h^{SNP}\left(t|z_{i}^{SNP}\right) &=h_{0}^{SNP}(t)\exp(\eta_{i}^{clin} + \eta_{i}^{SNP})\\ &=h_{0}^{SNP}(t)\exp\left(x_{i}^{'clin} \alpha^{SNP} + x^{'SNP}_{i} \gamma^{SNP}\right). \end{aligned}  $$

The hazard $h^{SNP}(t|z_{i}^{SNP})$ is the instantaneous risk of observing an event at time *t* given survival until *t* conditional on the clinical and SNP covariates. It is modeled by the unspecified baseline hazard $h_{0}^{SNP}(t)$ and the linear predictors $\eta _{i}^{clin}$ and $\eta _{i}^{SNP}$ for the clinical and SNP covariates with parameter vectors $\alpha ^{SNP}=(\alpha ^{SNP}_{1},\ldots,\alpha ^{SNP}_{d})'$ and $\gamma ^{SNP}=(\gamma _{1}^{SNP},\ldots,\gamma _{k}^{SNP})'$. Subsequently, the parameters *γ*^*SNP*^ will be estimated by a regularized approach for obtaining many estimates equal to zero and only a small set of signature SNPs with non-zero estimates. The parameters *α*^*SNP*^ are estimated using standard maximum (partial) likelihood techniques without penalization.

This Cox proportional hazard model for the SNP data can be used for guiding the fit of a Cox proportional hazard model for the microarray-based GEP data for identifying genes whose expression provides prognostic information beyond the SNP data. In particular, we propose to use regularized estimation for fitting a model

2 $$ {{\begin{aligned} {}h^{GEP}(t|z_{i}^{GEP}) = h_{0}(t)^{GEP}\exp\left({\hat{\eta}_{i}^{SNP} + x_{i}^{'clin}\alpha^{GEP} + x_{i}^{'GEP}\beta^{GEP}}\right), \end{aligned}}}  $$

where $h_{0}^{GEP}(t)$ is the unspecified baseline hazard and parameter vector $\beta ^{GEP}=(\beta _{1}^{GEP},\ldots,\beta _{q}^{GEP})'$. For extracting the complementary information from the GEP data by adjusting for the effects of the SNP data in the model for the GEP data, $\hat {\eta }_{i}^{SNP}$ is incorporated as a fixed offset obtained from the estimates in model (1) (see Fig. 1, step 2). Note that the parameter vector *α*^*GEP*^ for the clinical covariates is different from *α*^*SNP*^ in model (1), as it is now used to take the clinical covariates into account when considering gene expression instead of SNP measurements. In particular, we will use unregularized estimation, as detailed in the section on estimation below, for maximally adjusting for the effect of clinical effects in both prognostic models, i.e. in that based on the SNPs and also in that based on GEP measurements. The reason for this is that we are interested in information from the SNP and GEP data that is not already contained in clinical covariates.

### Imputation approach and linking model

To address a partial overlap, we propose imputation of the linear predictor $\hat {\eta }_{i}^{SNP}$, called SNP signature in the following. After values for this linear predictor have been imputed for individuals without SNP measurements all available observations can be used when fitting model (2).

To impute the information from the SNP microarray for the individuals with measurements from the microarray-based GEP data only, a linking model is fitted. Using the samples with measurements available from both sources, i.e. SNPs and GEP, we consider the SNP signature $\hat {\eta }_{i}^{SNP}$ as a continuous response in a regression model with gene expression features as covariates (see Fig. 1, step 1b). Established clinical predictors again receive a separate parameter vector, to be estimated unregularized, to adjust out any effect that might have been introduced by estimation of their effect in model (1).

Specifically, we propose a continuous response linear regression model of the form

3 $$ \hat{\eta}_{i}^{SNP}=x_{i}^{'clin}\alpha^{link}+x_{i}^{'GEP}\beta^{link} + \epsilon_{i},  $$

where *ε*_*i*_ is the error term. The parameter vector $\beta ^{link}=(\beta _{1}^{link},\ldots,\beta _{q}^{link})$ for the GEP covariates is different from *β*^*GEP*^ in model (2), as it now reflects the information that can be extracted from the GEP covariates for predicting the SNP signature $\hat {\eta }_{i}^{SNP}$. Using the estimated parameters from the model above, the SNP signature is predicted for the non-overlap samples and incorporated in model (2) for the GEP data (see Fig. 1, step 2).

Based on the imputation approach, the sequential complementary strategy does not have to assemble all molecular measurements into a single model. Therefore, compared to approaches which consider both molecular levels in parallel [12], the sequential strategy has the advantage to take specific characteristics of the different molecular levels into account, e.g. biological relations between SNP and microarray-based GEP data.

### Estimation by componentwise likelihood-based boosting

The parameters in all three regression models above, i.e. both risk prediction models (1) and (2) for the SNP and GEP data respectively, and in the linking model (3), will be estimated by a regularized approach to avoid overfitting and for selecting a small set of SNPs and genes with non-zero estimated effects. Specifically, we use a componentwise likelihood-based boosting approach, which is available for continuous endpoints [6] as well as for Cox proportional hazards models [2]. The details are provided in these references, and we provide only a brief description in the following.

Componentwise likelihood-based boosting starts with estimated parameter vectors equal to zero and builds up estimates using a stepwise algorithm with a potentially large number of boosting steps. To obtain sparse fits, as intended in high-dimensional data, only one element of the parameter vector is updated in each boosting step. To identify the element to be updated, a candidate model is fitted for each covariate in each boosting step, comprising only this covariate and a fixed offset that contains the information from the previous boosting steps. The parameters in these models are estimated by a penalized maximum (partial) likelihood approach, and finally the element to be updated is chosen to be the one that maximizes the (partial) log-likelihood. Componentwise likelihood-based boosting can distinguish between mandatory covariates, which are always included in the model, e.g. established clinical predictors that need to be adjusted for, and optional covariates. While the parameters for the optional covariates are estimated as described above, the parameters for the mandatory covariates are estimated by standard maximum (partial) likelihood techniques. For estimating the parameters in the three regression models (1), (2), and (3), the clinical covariates are treated as mandatory, while the SNP and GEP covariates are considered as optional, i.e. are subjected to regularization. The main tuning parameter of the boosting algorithm is the number of boosting steps, which is selected by cross-validation to optimize prediction performance and to avoid overfitting. The penalty parameter in the penalized (partial) likelihood is of minor importance, as long as it is large enough.

### Ordering of molecular levels for analysis

The proposed strategy integrates data from two molecular levels with potentially small overlap in the biological samples, while taking established clinical predictors into account. The sequential approach is not based on the assumption of direct links between molecular levels as both sources are used in different roles. The sequential order of the different data sources depends on the underlying question of the integrative analysis. Such an order can be based on the biological relations, such as between SNP and microarray-based GEP data or can be swapped, e.g. first a prognostic model for GEP data could be fitted, and the linear predictor from this model could then be used for adjustment in prognostic model building from the SNP microarray data. While this order could be considered to be contrary to biological relations, such analyses could nevertheless be performed. However, the sequential order should be prespecified before starting the integrative analysis, to avoid decision making based on the results.

For the first application with GEP and SNP data, the SNP microarray data are analyzed first and the results are used for guiding GEP features that contain information beyond what is already provides by the SNPs in the following, reflecting the biological relation between SNP and GEP data. For the second application, investigating gene expression sequencing (RNA-seq) and methylation data, we consider RNA-seq data first and then analyze the methylation data adjusted for RNA-seq effects, thus potentially better highlighting the signals in the methylation data that are not directly visible in the RNA-seq measurements.

## Results

### Application for combining SNP and GEP data

In a first application, we illustrate the proposed approach for a complementary integrative analysis of SNP and microarray-based GEP data from AML patients with respect to relapse free survival. As a reference approach we fit a risk prediction model for the GEP data without adjusting for the effects of the SNP data. The model for the reference approach will be estimated again by componentwise likelihood-based boosting. As criteria for comparison, we will consider stability of selected signatures, as quantified by resampling inclusion frequencies, as well as prediction performance.

#### Model building

For modeling the effects of the SNPs, we use a coding scheme with two dummy covariates per SNP to distinguish between dominant and recessive effects. Minor allele frequencies are coded as $z^{(1)}_{i_{(j)}}=0$ and $z^{(1)}_{i_{(j+1)}}=1$ for 0, $z^{(1)}_{i_{(j)}}=1$ and $z^{(1)}_{i_{(j+1)}}=0$ for 1 and $z^{(1)}_{i_{(j)}}=1$ and $z^{(1)}_{i_{(j+1)}}=1$ for 2 (see Reference [13] for example), *i*=1,…,308 and *j*=1,…,390443. Shrunken estimates, as obtained by the boosting technique, then take the meaning of ’no/small’-dominant and ’no/small’ recessive effects. The predictors age, WBC, *NPM*1 and *FLT*3−*ITD* status as well as a cytogenetic risk group factor are included as mandatory covariates in addition to the optional SNP covariates in componentwise likelihood-based boosting used to obtain sparse fits from the SNP data.

Twenty-three SNPs are selected for the SNP risk prediction model (1), i.e. they received non-zero estimates. The estimated coefficient paths of these SNPs are given in Additional file 3. The vertical line indicates the optimal number of boosting steps as selected via 10-fold cross-validation. For all subsequent uses of boosting, the number of boosting steps was also selected via 10-fold cross-validation. The resulting SNP signature ($\hat {\eta }_{i}^{SNP}$) includes the most important prognostic information from the SNP microarray for each of the 308 biological samples with measurements available from this molecular level.

To address the partial overlap in the biological samples, imputation of the SNP signature is performed by fitting linking model (3) for the 26 biological samples using the GEP features conditioning on the overlap samples. The same five clinical predictors for adjustment as in risk prediction model (1) for the SNP data are used as mandatory covariates in addition to the optional GEP covariates. Eight GEP features are selected for the linking model (3) by boosting with respect to the continuous endpoint, i.e. the SNP signature. The coefficient paths of these GEP features are shown in Additional file 3. Next, imputation by prediction of the SNP signature is done for the 293 AML patients with measurements from the microarray-based GEP only.

To judge the model fit on the overlap samples the mean squared error is determined by the bootstrap.632+ technique [14], which is a weighted combination of the apparent error and the bootstrap cross-validation estimate. The resulting prediction error estimate of 0.13 does not indicate particularly good performance. However, it has to be considered that the model is based on 26 overlap samples only and does perform only slightly worse than imputing the mean value of the SNP signature (prediction error of 0.11 determined by the apparent error). We nevertheless evaluate in the following whether the resulting imputed linear predictor might prove useful for adjustment when considering effects of GEP measurements on relapse-free survival.

For extracting complementary information from the GEP data, the SNP signature is incorporated as a fixed offset into the risk prediction model (2) using the GEP data. Risk prediction model (2) is estimated by componentwise likelihood-based boosting incorporating the same five mandatory clinical predictors as in risk prediction model (1) and linking model (3). While thirty-four GEP features are selected by the sequential complementary strategy, the reference approach selects twenty-seven GEP features. The estimated coefficient paths for the GEP covariates selected by the sequential complementary strategy and the reference approach, respectively, are shown in Additional file 4. Twenty-one of the twenty-seven GEP features selected by the reference approach are also within the thirty-four GEP features selected by the sequential complementary strategy. This is, the remaining thirteen GEP features of the thirty-four GEP features obtained by the sequential complementary strategy are selected only when incorporating the (imputed) linear predictor of the SNP signature as fixed offset in the risk prediction model together with the GEP data. Therefore, complementary information can be extracted from the gene expression measurements after using the effects from the SNP data for guiding the GEP data.

Some IMAGE (Integrated Molecular Analysis of Genomes and their Expression) cDNA clones are not linked to genes, but rather represent expressed sequence tags (ESTs), whereas other IMAGE clones represent annotated genes. Note that single genes can be represented by several IMAGE clones, similar to Affymetrix arrays where single genes are often covered by numerous probe sets.

#### Variable selection stability

Stability quantified by resampling techniques is considered to judge model quality. The underlying idea is to perform model building (including variable selection) in several resampling data sets and to consider for each covariate the proportion of final models derived from the resampled data sets where it has been selected. See for example [15, 16] for more details of quantifying signature stability by resampling inclusion frequencies. However, stability criteria have also been applied to enhance and to improve existing methods for variable selection by supporting consistent structure estimation and optimizing variable selection methods called stability selection [17]. Selection stability has even be suggested obtaining the false discovery rates [17]. We rather use stability as a criterion for comparing the sequential complementary strategy and the reference approach to investigate model structure and to draw inferences from the statistical strategy on the relation between the different molecular sources. Therefore, the aim of this work is not to optimize neither the sequential complementary strategy nor the established risk prediction model used as reference approach. Stability of a selected model is used as an evaluation criterion to judge a given variable selection procedure or to compare different variable selection procedures [15].

In the following, feature selection of the molecular entities is performed for each of 100 resampling data sets, i.e. the sequential complementary strategy as well as the reference approach are applied. Resampling can be performed with replacement, corresponding to the bootstrap, or without replacement, i.e. subsampling. However, resampling with replacement introduces a bias that will affect approaches that select a tuning parameter, e.g. the number of boosting steps in each resampling data set, which is selected according to prediction performance to avoid overfitting using 10-fold cross-validation. See Reference [18] for more details on this bias. Therefore, we use subsampling in the following for generating data sets of size 0.632*n*. The resampling inclusion frequency algorithm is illustrated in Additional file 5.

**Fig. 2:**
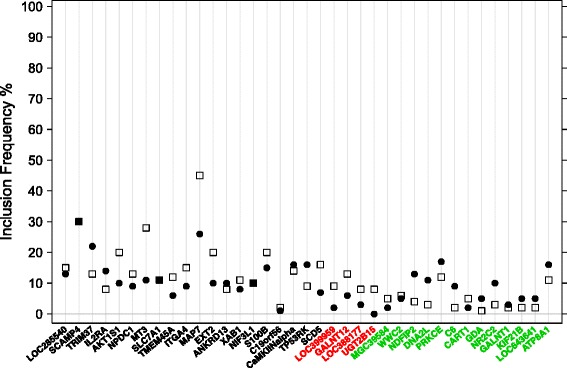
Resampling inclusion frequencies for the genes selected by the sequential as well as by the reference approach (*black*), genes selected only by the reference approach (*red*) and genes selected only by the sequential complementary strategy (*green*) from the GEP data (first AML application). The inclusion frequencies for these genes concerning the reference approach are displayed by squares and the inclusion frequencies for these genes concerning the sequential complementary strategy are displayed by dots. Reference approach and sequential complementary strategy are estimated by componentwise boosting

In the first AML application, we consider the inclusion frequency for each gene selected in the original GEP data by the sequential complementary strategy (33 genes compared to 34 IMAGE clones) and by the reference approach (24 genes compared to 27 IMAGE clones) shown in Fig. 2. Genes selected by the sequential complementary strategy as well as by the reference approach in the original GEP data are displayed in black, genes selected by the reference approach only are displayed in red and genes selected by the sequential complementary strategy only are displayed in green. Figure 2 shows a varying inclusion frequency for these genes selected from both strategies in the original GEP data. There are at least some genes selected by both strategies that have an increased inclusion frequency in the sequential strategy. For some other genes there is a decreased inclusion frequency. As an underlying pattern, genes that are selected in the original GEP data by the reference approach only (indicated by red) have a systematically smaller inclusion frequencies when using the sequential complementary strategy. Reversely, genes that are selected on the original GEP data by the sequential complementary strategy also have increased inclusion frequencies when using this approach. There are even four genes selected only by the sequential complementary strategy in the original data which received an inclusion frequency greater than 10 % only when using the sequential complementary strategy. These systematic differences in the inclusion frequencies indicate that different lists of genes are stably selected when using the reference and the sequential complementary approach, respectively.

Specific components of the sequential complementary strategy, such as boosting, can be replaced by other modeling techniques. In particular, by penalized regression approaches, such as lasso [1]. To carry out a comparison of the results obtained from the sequential complementary strategy based on boosting and other modeling approaches, the risk prediction model (2) for the microarray-based GEP data was also estimated by the lasso. Within the stepwise procedure of the sequential strategy, risk prediction models (1) and (3) will still be estimated by componentwise boosting. Nineteen genes are selected by the reference approach based on lasso and twenty-eight genes are selected by the sequential complementary strategy where risk prediction model (2) is estimated by lasso. The inclusion frequency patterns for the genes are very similar to those resulting from componentwise boosting (Additional file 6).

#### Prediction performance

To investigate whether the sequential complementary strategy provides reasonable prediction performance and to additionally verify that the extracted complementary information from the GEP measurements after adjusting for the effects from the SNP microarray is not only due to noise, the prediction performances of the sequential and reference approach are investigated. For evaluating the prediction performance, the Brier score [19], i.e. a time-dependent measure of prediction error, is used resulting in a prediction error curve. Prediction error estimates are obtained by the bootstrap.632+ technique [18, 20, 21]. As a conservative reference for prediction performance the Kaplan-Meier benchmark that does not use any covariate information is used. The performances of the sequential and the reference approach are furthermore compared to a Cox model that includes only the five clinical predictors as in risk prediction models (1), (2) and (3).

**Fig. 3:**
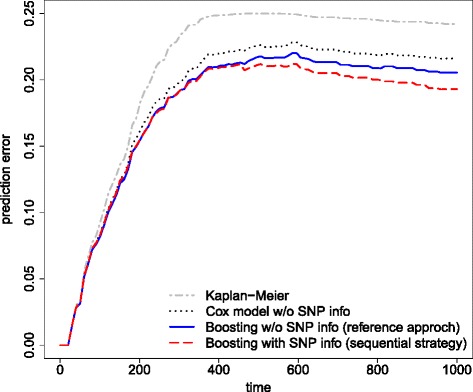
Prediction error curves for the first AML data application example. Bootstrap.632+ prediction error curves estimates for sequential complementary strategy, i.e. boosting including SNP information for adjustment (*dashed red curve*) and for the reference approach, i.e. boosting without including SNP information (*solid blue curve*). The Kaplan-Meier benchmark is indicated by the dashed-dotted gray curve and the Cox model is given by dotted black curve

Figure 3 shows the.632+ prediction error estimates for the sequential complementary strategy (dashed red curve) and the reference approach (solid blue curve). In addition, the estimated prediction error curve for the Cox model, that includes only the five important clinical predictors (dotted black curve), is displayed. The Kaplan-Meier benchmark (grey dashed-dotted curve) is given as reference. All prediction error curve estimates are seen to improve over the Kaplan-Meier benchmark. The prediction performances of the sequential complementary strategy as well as of the reference approach also improve over the Cox model. It is seen that the performance of the sequential complementary strategy is roughly similar to prediction performance of the reference approach at early times. However, the sequential complementary strategy seems to improve over the reference approach for later time points. Thus, the imputed linear predictor seems to contain useful information.

**Fig. 4:**
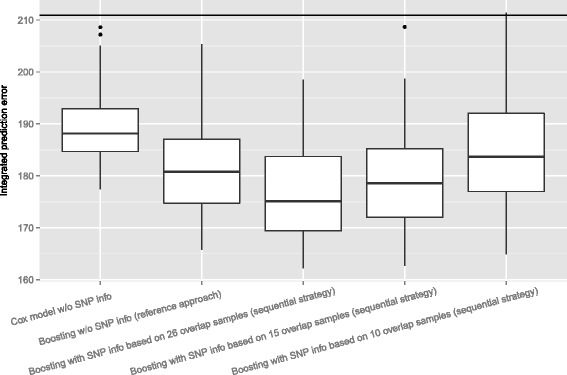
Variability of the.632+ prediction error estimates in the first AML application based on varying overlap sizes of 26, 15 and 10 biological samples, respectively. Integrated prediction error curve estimates for the Cox model, the reference approach and the sequential complementary strategy. The performance of the Kaplan-Meier benchmark is indicated by a horizontal line

Figure 4 shows boxplots of the integrated prediction error curves, i.e. the.632+ prediction error estimate is calculated separately for each single bootstrap sample to evaluate the variability of the prediction performance underlying the estimates in Fig. 3. The conclusions drawn from Fig. 3 hold, even when variability is taken into account. The sequential complementary strategy based on 26 overlap samples implies better prediction performance compared to the reference approach and both outperform the Kaplan-Meier benchmark as well as the Cox model.

#### Effect of the overlap size

One might expect that the relative size of overlap has a considerable impact on the performance of the sequential complementary strategy. Therefore, in the first AML application, we systematically decreased the size of overlap from 26 to 10 biological samples. This is done by randomly drawing 15 and 10 biological samples, respectively, from the 26 overlap samples with available measurements from both molecular data in parallel. The remaining biological samples, which are not randomly drawn, are treated as if they would have measurements from the GEP data only. Figure 4 shows the resulting prediction performance. For 15 overlap samples there is still a reasonable performance still implying better prediction performance compared to the reference approach. However, an overlap size of 10 biological samples results in very unfavorable prediction performance compared to the performance of the reference approach and of the sequential strategy based on 26 and 15 biological samples, respectively.

In addition, to examine the effect of the overlap size on the performance of the linking model (3) used for data imputation, the mean squared error is determined by the bootstrap.632+ technique. The resulting prediction error estimates for the varying overlap sizes are displayed in Additional file 7. The prediction performance of the null model, i.e. imputing the mean value of the SNP signature, determined by the apparent error is considered as a reference. Generally, the linking model (3) does not exhibit good prediction performance. In particular, for all varying overlap sizes the linking model results in performance worse than the intercept model. Nevertheless, the resulting imputation still enables somewhat improved prediction performance with respect to the clinical endpoint, as seen in Fig. 4.

#### Different order of molecular levels

A swapped order of molecular levels within the sequential complementary strategy is investigated in the first AML application by analyzing first the GEP data and using the corresponding linear predictor for adjustment in a risk prediction model based on the SNP microarray data. Boxplots of the resulting integrated prediction error estimates are shown in Additional file 8. There also is a slight improvement in prediction performance of the sequential complementary strategy (swapped order) compared to the reference approach, i.e. SNP model without including GEP information for adjustment. Thus, there is no firm conclusion with respect to biological relations. Correspondingly, the more pronounced improvement in prediction performance when using the biologically more plausible order in the sequential strategy (Fig. 4) might not just be due to chance.

### Application for combining methylation and gene expression sequencing (RNA-seq) data

**Fig. 5:**
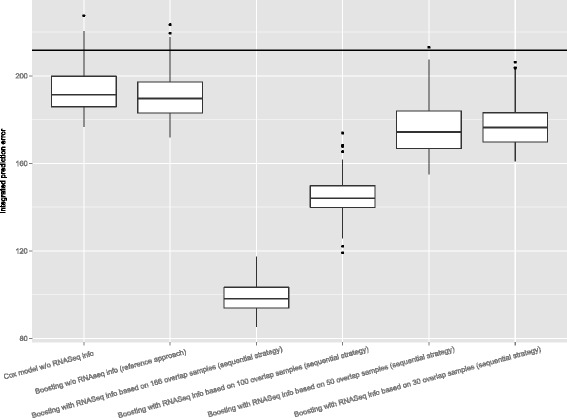
Variability of the.632+ prediction error estimates in the second AML application based on varying overlap sizes of 166, 100, 50 and 30 biological samples, respectively. Integrated prediction error curve estimates for the Cox model, the reference approach and the sequential complementary strategy (verification example). The performance of the Kaplan-Meier benchmark is indicated by a horizontal line

In the second AML application, we consider integration of methylation and gene expression sequencing measurements with respect to overall survival. For the methylation data, we use M-values, which are simply a transformation of the beta value [22]. The latter is a number between zero and one that measures the percentage of methylation. The RNA-seq data are normalized using the DESeq normalization proposed by [23]. The measurements are log-transformed, following the suggestion in [24]. In the following, the sequential order is chosen such as to first extract information from the RNA-seq measurements and then to use the results to better highlight remaining signals in the methylation data. There is an overlap of about 92 % in the biological samples. Imputation of the RNA-seq signature is performed by fitting a linking model (3) using methylation data conditioning for 166 overlap samples. The resulting prediction error estimate of 0.25 determined by the bootstrap.632+ technique does indicate good prediction performance as the linking model improves the benchmark model by imputing the mean value of the RNA-seq signature (prediction error of 0.93 determined by the apparent error). In addition, the effect of the size of overlap on the performance of the sequential complementary strategy is investigated by systematically decreasing the size from about 92 % to about 18 %. Specifically, this is done by drawing 100, 50 and 30 biological samples, respectively, from the 166 overlap samples. Even more pronounced as in the first application, the prediction performance of the sequential complementary strategy improves over the reference approach (Fig. 5). In particular, a considerable advantage remains even for rather small overlap sizes.

## Discussion

Recently, a multitude of techniques have become available to obtain genome-wide data sets at various molecular levels. Integrative analysis of such molecular data from different platforms might provide additional insight. For example, our motivation for the integrative analysis of various molecular levels came from an application with AML patients, where there is still huge unexplained heterogeneity within the defined molecular subtypes based on clinically relevant molecular marker from single genetic data. Our aim was to develop a strategy for integrating multiple genome-wide data sets in a complementary way, despite a potentially small overlap in the biological samples. This also is a potential alternative to parallel approaches which incorporate all information in once but require availability of all sources for all individuals. In the first AML application, we considered measurements SNP microarrays and microarray-based gene expression (GEP) measurement. Both molecular data sets need to be combined into an integrative data analysis for identifying prognostic signatures. In the second application, we considered integrative analysis for gene expression sequencing (RNA-seq) and methylation measurements.

We developed a sequential complementary strategy that is a stepwise procedure based on componentwise likelihood-based boosting. The stepwise procedure analyzes first one molecular level individually and then the results from the first molecular data set are used for guiding the model for another molecular data set. Specifically, this means that the sequential complementary strategy is designed for extracting complementary information from the second molecular data set by adjusting for the effects from the first molecular level in the model for the second data set. The developed strategy is not based on the assumption of direct links between molecular levels. Such a sequential complementary strategy can take the biological hierarchies between the different molecular levels into account, e.g. focusing on GEP features that provide information beyond SNPs, or the order can be swapped. Also, more than two sources could potentially be investigated by considering several linear predictors.

To assess the proposed sequential complementary strategy we considered a reference approach, where a prognostic model was fitted based on the GEP data without adjusting for the effects of the SNP microarray data in the first application. Twenty genes were identified by the sequential as well as by the reference approach. In addition to the twenty genes, thirteen genes were identified by the sequential complementary strategy only. This might be an instance where the sequential complementary strategy provides molecular features that were not selected by a risk prediction model considering a single molecular data set only. When considering resampling inclusion frequencies for judging signature stability, genes selected by the sequential complementary strategy in the original GEP data also received larger inclusion frequencies when using this strategy, as compared to the reference approach, further underlining that our strategy results in a structurally different stable set of prognostic genes.

Importantly, in the first application, the list of genes identified from the GEP data after adjusting for the effects from the SNP data was biologically meaningful including both genes previously reported to be associated with outcome in AML such as *DNAL2* [4]. Similarly, *NDFIP2* represents another interesting leukemia relevant candidate gene as it is an important element of the ubiquitin-proteasome system (UPS) that plays an essential role in the homeostasis of cellular protein traffic and degradation, regulating cell fate, together with autophagy and apoptosis. While disruption of UPS is essential for leukemogenesis, recently *NDFIP2* was found deregulated and associated with blast counts in AML [25]. In addition, the list contained novel candidates so far not linked to AML in more detail, such as, e.g. *NR2C2* (nuclear receptor subfamily 2, group C, member 2) coding for a ligand-activated transcription factor that is an important repressor of nuclear receptor signaling pathways such as retinoic acid receptor, which is crucial for the differentiation of myeloid cells. Similarly, the *PRKCE* (protein kinase C, epsilon) gene represents an interesting candidate belonging to the serine- and threonine-specific protein kinases that phosphorylate a wide variety of protein targets and are known to be involved in diverse cellular signaling pathways also important in the differentiation of myeloid cells.

In the second AML application, improved prediction performance compared to a reference approach indicated that the proposed imputation strategy works well for different overlap sizes, in particular still providing some gain even for a small overlap.

## Conclusion

The sequential strategy was developed to resolve the problem of the complementary integrative analysis of different molecular data sets with potentially partial overlap in the biological samples. It provided promising results in two applications to data from AML patients. While we explored it in the context of these specific applications, it does not depend on these examples and can also be applied to similar problems. Specific components within the stepwise procedure, such as componentwise boosting, might be replaced by other modeling approaches, e.g. lasso, thus providing a more general strategy. However, such modifications need further research in the future. Naturally, as the results from the AML application have only exemplary character, further research is needed for refining and validating of the proposed approach to arrive at such a more general strategy. However, the present results already indicate that such an approach might be promising.

## Additional files

Additional file 1 Single nucleotide polymorphism (SNP) microarray data. SNP data underlying the finding in this article. (Rdata 50688 kb)

Additional file 2 Microarray-based gene expression profiling (GEP) data. GEP data underlying the finding in this article. (Rdata 44977 kb)

Additional file 3 Coefficient paths of SNP and GEP measurements (first AML application example). Parameter estimates obtained from componentwise likelihood-based boosting for the SNP microarray data (left panel) and parameter estimates obtained from componentwise likelihood-based boosting for the microarray-based GEP data conditioning on the overlap samples (right panel), plotted against the number of boosting steps. (PDF 64 kb)

Additional file 4 Coefficient paths of the GEP features selected from the sequential complementary strategy and from the reference approach (first AML application example). Parameter estimates obtained from sequential complementary strategy for the microarray-based GEP data (left panel) and parameter estimates obtained from the reference approach for the microarray-based GEP data (right panel), plotted against the number of boosting steps. (PDF 74 kb)

Additional file 5 Resampling inclusion frequency algorithm. Resampling inclusion frequency setup investigated to evaluate model stability. (PDF 77 kb)

Additional file 6 Resampling inclusion frequency patterns based on lasso (first AML application example). Resampling inclusion frequencies for the genes selected by the sequential as well as by the reference approach (black), genes selected only by the reference approach (red) and genes selected only by the sequential complementary strategy (green) from the GEP data (first AML application example). The inclusion frequencies for these genes concerning the reference approach are displayed by squares and the inclusion frequencies for these genes concerning the sequential complementary strategy are displayed by dots. Reference approach and risk prediction model (2) within the sequential complementary strategy are estimated by lasso. (PDF 36 kb)

Additional file 7 Boxplots of bootstrap 632+ prediction error estimates of the linking model for varying overlap sizes (first AML application example). Boxplots of bootstrap 632+ prediction error estimates of the linking model (3) from single resampling data sets conditioning on the decreased overlap sizes. (PDF 34 kb)

Additional file 8 Variability of the.632+ prediction error estimates in the first AML data application example (swapped order). Boxplots of the integrated prediction error curve estimates for the Cox model, the SNP model and the sequential complementary strategy (swapped order). (PDF 35 kb)

Additional file 9 R code. This R-file provides the underlying R code to reproduce the results of the first AML application example combining SNP and microarray-based gene expression data. (R 33 kb)

## Abbreviations

AML: Acute myeloid leukemia
clin: Clinical predictors
CpG: Cytosine-phosphate-guanine sites
*FLT*3−*ITD*: Fms-related tyrosine kinase 3 internal tandem-duplication
GEP: Gene expression profiling
link: Linking model
*NPM*1: Nucleophosmin 1 gene
OS: Overall survival
RFS: Relapse free survival
RNA-seq: RNA sequencing
SNP: Single nucleotide polymorphism
TCGA: The Cancer Genome Atlas data portal
WBC: White blood cell count
