# Supplementary material for: Integrating multiple molecular sources into a clinical risk prediction signature by extracting complementary information
Source: BMC Bioinformatics. 2016 Aug 30;17(1):327. doi: 10.1186/s12859-016-1183-6 (PMC5004308; doi:10.1186/s12859-016-1183-6)
Supplement: Additional file 3 — Coefficient paths of SNP and GEP measurements (first AML application example). Parameter estimates obtained from componentwise likelihood-based boosting for the SNP microarray data (left panel) and parameter estimates obtained from componentwise likelihood-based boosting for the microarray-based GEP data conditioning on the overlap samples (right panel), plotted against the number of boosting steps. (PDF 64 kb) [file 12859_2016_1183_MOESM3_ESM.pdf]

Coefficient paths of SNP and GEP measurements
(first AML application example)

(a) Coefficient paths of the selected SNPs

(b) Coefficient paths of the selected gene expression features

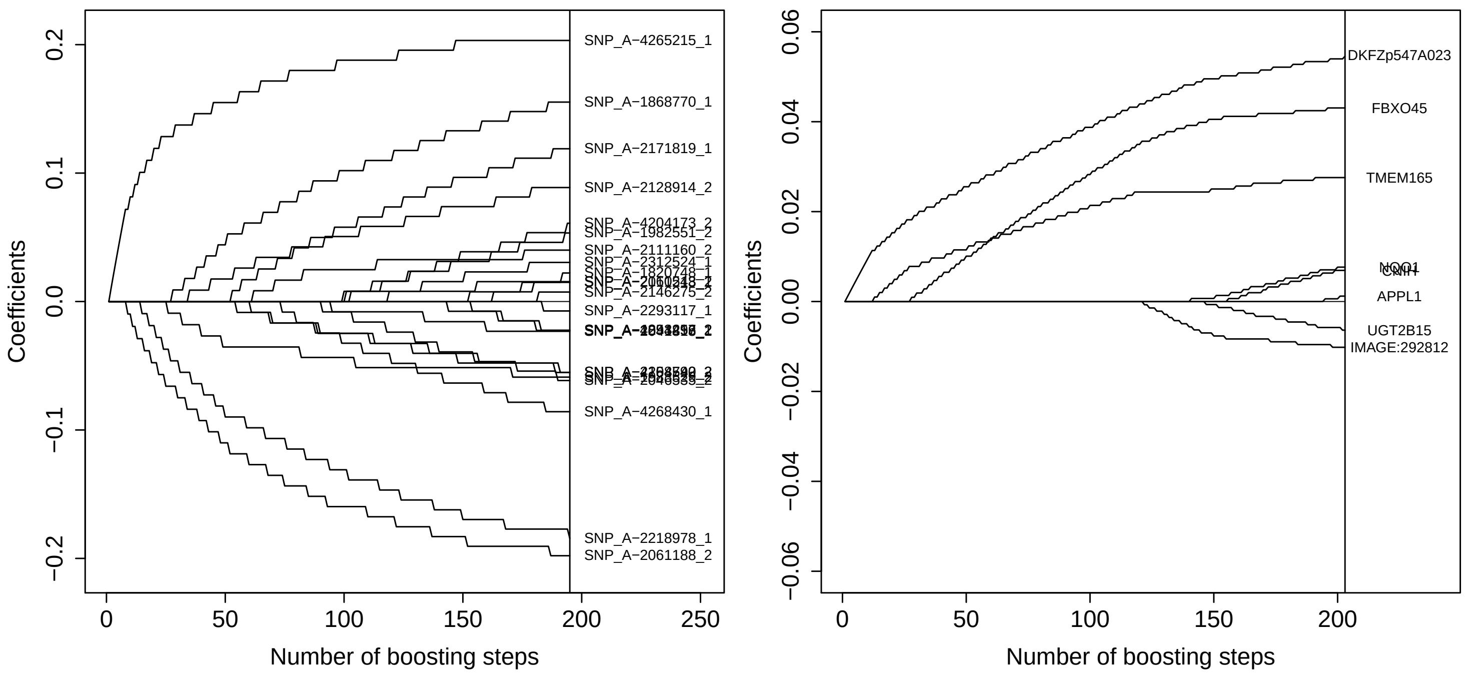

Parameter estimates obtained from componentwise likelihood-based boosting for the SNP microarray data (left panel) and parameter estimates obtained from componentwise likelihood-based boosting for the microarray-based GEP data conditioning on the overlap samples (right panel), plotted against the number of boosting steps. The optimal number of boosting steps is 194 for the SNP data and 202 for the GEP data conditioning on the overlap samples.
