# Supplementary material for: Integrating multiple molecular sources into a clinical risk prediction signature by extracting complementary information
Source: BMC Bioinformatics. 2016 Aug 30;17(1):327. doi: 10.1186/s12859-016-1183-6 (PMC5004308; doi:10.1186/s12859-016-1183-6)
Supplement: Additional file 4 — Coefficient paths of the GEP features selected from the sequential complementary strategy and from the reference approach (first AML application example). Parameter estimates obtained from sequential complementary strategy for the microarray-based GEP data (left panel) and parameter estimates obtained from the reference approach for the microarray-based GEP data (right panel), plotted against the number of boosting steps. (PDF 74 kb) [file 12859_2016_1183_MOESM4_ESM.pdf]

Coefficient paths of the GEP measurements selected from the sequential complementary strategy and from the reference approach (first AML application example)

(a) Coefficient paths of the selected features from the sequential strategy (b) Coefficient paths of the selected features from the reference approach

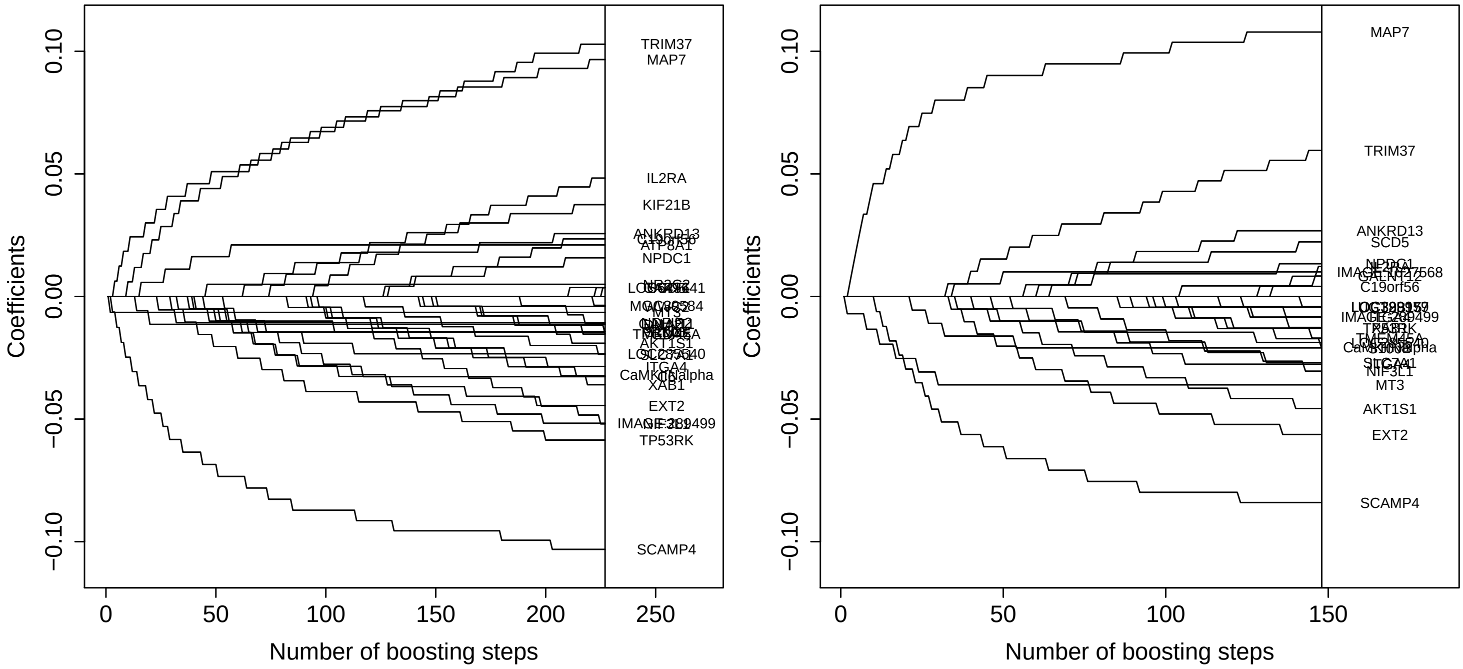

Parameter estimates obtained from sequential complementary strategy for the microarray-based GEP data (left panel) and parameter estimates obtained from the reference approach for the microarray-based GEP data (right panel), plotted against the number of boosting steps. The optimal number of boosting steps is 226 for the sequential complementary strategy and 147 for the reference approach.
