# Supplementary material for: Integrating multiple molecular sources into a clinical risk prediction signature by extracting complementary information
Source: BMC Bioinformatics. 2016 Aug 30;17(1):327. doi: 10.1186/s12859-016-1183-6 (PMC5004308; doi:10.1186/s12859-016-1183-6)
Supplement: Additional file 5 — Resampling inclusion frequency algorithm. Resampling inclusion frequency setup investigated to evaluate model stability. (PDF 77 kb) [file 12859_2016_1183_MOESM5_ESM.pdf]

Resampling inclusion frequency algorithm

$*$ Repeat for resampling data set $b = 1, \ldots, B$:

1. A random sample without replacement of size $0.632n$ is drawn from the complete observations $1, \ldots, n$ and assigned to subsample b.

2. Apply a variable selection procedure using subsample b:

   (a) For each molecular feature z_j $(j = 1, \ldots, q)$ determine whether z_j is selected in the model.

   (b) The parameter indices of the selected molecular features are taken to be in the set $J_{S_b} \subset \{1, \ldots, q\}$.

   (c) For each molecular feature index $j \in \{1, \ldots, q\}$, compute the indicator $\rho_{j_b} = I(j \in J_{S_b})$ with $I(.)$ being the indicator function

$$I(j \in J_{S_b}) = \begin{cases} 1, & \text{if} \quad j \in J_{S_b} \\ 0, & \text{if} \quad j \notin J_{S_b} \end{cases}$$

   $\Rightarrow$ ρ_{j_b} taking the value 1 if z_j was selected in the bth subsample.

$*$ Assess the resampling inclusion frequency (IF):

For each molecular feature z_j $(j = 1, \ldots, q)$, the resampling inclusion frequency is given by

$$\text{IF}(z_j) = \sum_{b=1}^{B} \rho_{j_b}$$
