# Supplementary material for: Integrating multiple molecular sources into a clinical risk prediction signature by extracting complementary information
Source: BMC Bioinformatics. 2016 Aug 30;17(1):327. doi: 10.1186/s12859-016-1183-6 (PMC5004308; doi:10.1186/s12859-016-1183-6)
Supplement: Additional file 6 — Resampling inclusion frequency patterns based on lasso (first AML application example). Resampling inclusion frequencies for the genes selected by the sequential as well as by the reference approach (black), genes selected only by the reference approach (red) and genes selected only by the sequential complementary strategy (green) from the GEP data (first AML application example). The inclusion frequencies for these genes concerning the reference approach are displayed by squares and the inclusion frequencies for these genes concerning the sequential complementary strategy are displayed by dots. Reference approach and risk prediction model (2) within the sequential complementary strategy are estimated by lasso. (PDF 36 kb) [file 12859_2016_1183_MOESM6_ESM.pdf]

Resampling inclusion frequency patterns based on lasso (first AML application example)

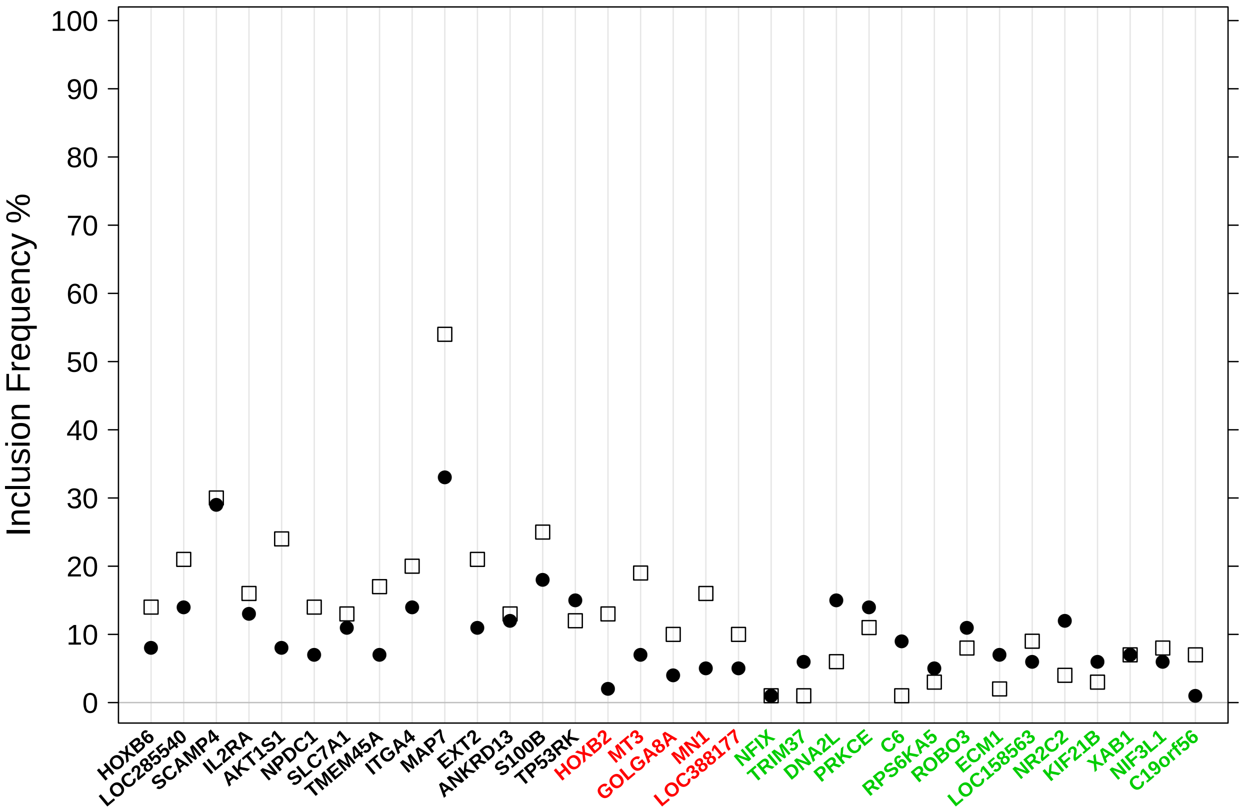

Resampling inclusion frequencies for the genes selected by the sequential as well as by the reference approach (black), genes selected only by the reference approach (red) and genes selected only by the sequential complementary strategy (green) from the GEP data (first AML application example). The inclusion frequencies for these genes concerning the reference approach are displayed by squares and the inclusion frequencies for these genes concerning the sequential complementary strategy are displayed by dots. Reference approach and risk prediction model (2) within the sequential complementary strategy are estimated by lasso.
