# Supplementary material for: Integrating multiple molecular sources into a clinical risk prediction signature by extracting complementary information
Source: BMC Bioinformatics. 2016 Aug 30;17(1):327. doi: 10.1186/s12859-016-1183-6 (PMC5004308; doi:10.1186/s12859-016-1183-6)
Supplement: Additional file 7 — Boxplots of bootstrap 632+ prediction error estimates of the linking model for varying overlap sizes (first AML application example). Boxplots of bootstrap 632+ prediction error estimates of the linking model (3) from single resampling data sets conditioning on the decreased overlap sizes. (PDF 34 kb) [file 12859_2016_1183_MOESM7_ESM.pdf]

# Boxplots of bootstrap 632+ prediction error estimates of the linking model for varying overlap sizes (first AML application example)

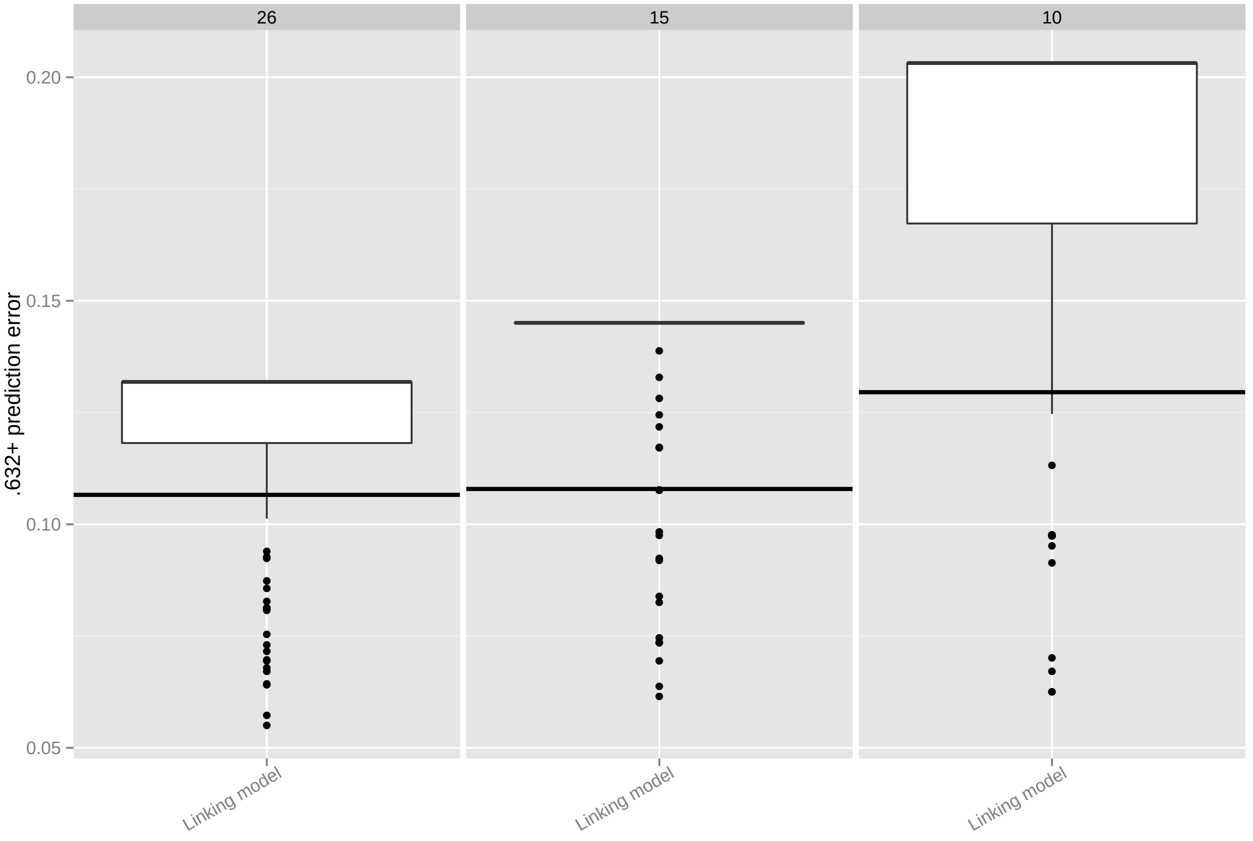


Boxplots of bootstrap 632+ prediction error estimates of the linking model (3) from single resampling data sets conditioning on the decreased overlap sizes. Corresponding to the varying size of overlap, the benchmark null model estimated by the apparent error is indicated by horizontal lines.
