# Supplementary material for: Integrating multiple molecular sources into a clinical risk prediction signature by extracting complementary information
Source: BMC Bioinformatics. 2016 Aug 30;17(1):327. doi: 10.1186/s12859-016-1183-6 (PMC5004308; doi:10.1186/s12859-016-1183-6)
Supplement: Additional file 8 — Variability of the.632+ prediction error estimates in the first AML data application example (swapped order). Boxplots of the integrated prediction error curve estimates for the Cox model, the SNP model and the sequential complementary strategy (swapped order). (PDF 35 kb) [file 12859_2016_1183_MOESM8_ESM.pdf]

# Variability of the .632+ prediction error estimates in the first AML data application example (swapped order)

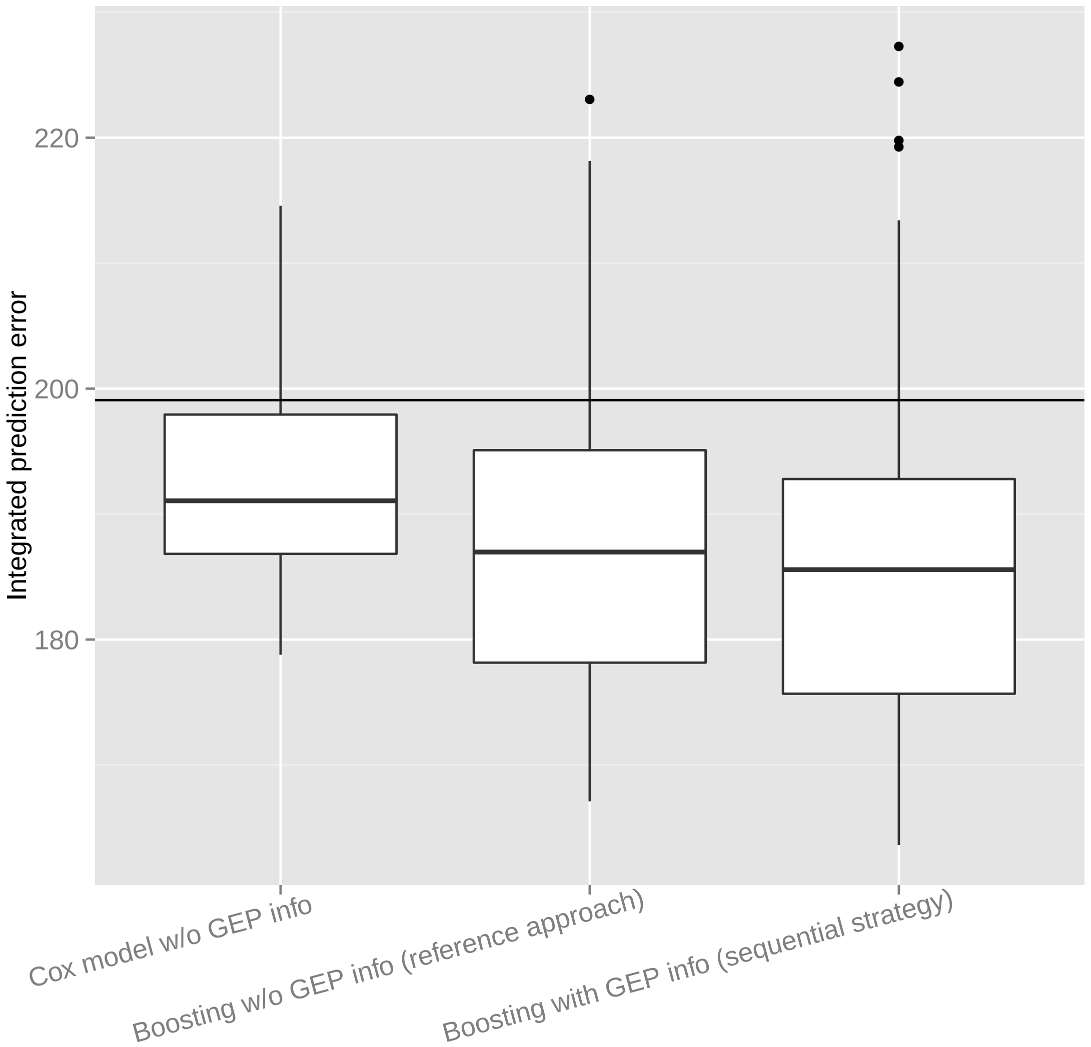


Boxplots of the integrated prediction error curve estimates for the Cox model, the SNP model and the sequential complementary strategy (swapped order). The performance of the Kaplan-Meier benchmark is indicated by a horizontal line.
